# Assessing the Impact of Computable Type 2 Diabetes Phenotypes on Predicting Healthcare Utilization Using Electronic Health Records and Administrative Claims

**DOI:** 10.3390/healthcare13182292

**Published:** 2025-09-12

**Authors:** Priyanka D. Sood, Star Liu, Chintan Pandya, Hadi Kharrazi

**Affiliations:** 1 Johns Hopkins School of Public Health, Baltimore, MD 21205, USA; pdua1@alumni.jh.edu (P.D.S.); cpandya2@jhu.edu (C.P.); 2 Johns Hopkins School of Medicine, Johns Hopkins University, Baltimore, MD 21205, USA; sliu197@jhmi.edu

**Keywords:** computational phenotypes, type 2 diabetes, population health management, risk stratification, predictive performance variation, healthcare utilization

## Abstract

**Background/Objectives**: Type 2 Diabetes (T2D) computable phenotypes, which leverage electronic health records (EHRs) and administrative claims data, provide the basis for T2D population health research. Our study investigates how T2D phenotypes affect downstream healthcare utilization prediction, specifically inpatient (IP) and emergency room (ER) admissions. **Methods**: This study included 15,338 adult patients from a large academic medical center with both EHR and claims data from 2017 to 2019. We compared widely adopted and locally developed T2D phenotypes. EHR predictors and claims-based outcomes were used for utilization prediction. Models were developed using a 70/30 training-and-test split over 100 iterations. Mean area under the curve (AUC), odds ratios (ORs), positive predictive values, and negative predictive values were reported. **Results**: Models had comparable performance in concurrent predictions. Impact of phenotypic variation impact was more apparent in prospective predictions. The CMS Chronic Conditions Data Warehouse (CCW) phenotype was more discriminatory in predicting concurrent IP and ER admissions (AUCs of 0.80 and 0.74) than prospective IP and ER visits (0.70 and 0.73) in 2019. **Conclusions**: Our study demonstrated how phenotypic variations and data sources impact healthcare utilization prediction in T2D patients. Furthermore, we highlight the significance of phenotype selection for targeted T2D population health initiatives and management strategies.

## 1. Introduction

Publicly available computable phenotype definitions embody the complexity of defining and identifying disparate denominator populations given varying data characteristics [1]. Computable phenotypes, also known as reusable algorithmic search queries, are often used to identify populations of patients with a similar condition or disease within large clinical data sources such as insurance claims or electronic health records (EHRs) [1]. Computable phenotypes of chronic diseases enable population health experts to formulate a standardized query to identify and extract patients with specific chronic conditions from a large EHR or claims database for analytic or interventional purposes [2].

Computable phenotypes are essential in identifying patients with similar conditions or diseases in large clinical data warehouses; however, gold-standard computable phenotypes are often missing for major chronic conditions [2,3]. For example, despite having multiple phenotype definitions, a commonly agreed phenotype for Type 2 Diabetes (T2D) is lacking in the risk stratification community. Indeed, despite the growing burden of T2D [4], significant variations exist in identifying T2D cases at a population level using common clinical data sources such as insurance claims or EHRs. Consequently, the American Diabetes Association [5] and the Healthy People 2030 collaboration [6] have emphasized the importance of understanding the variations in phenotypes in improving disease management and quality of care for patients with T2D.

Variations in computable phenotypes significantly impact the identification of population denominators used for population health analytics and management efforts [3,7]. Similarly, variations in phenotypes can also complicate the prediction of healthcare utilization. Models trained and tested on different T2D population denominators, which are identified using different phenotypes, may predict different groups of patients as high utilizers. These complications may lead to increased financial burden and contribute to disparities in identifying high-risk T2D patients within specific population groups. Such challenges further contribute to bias in predictive models, thus affecting their generalizability across populations identified using different computable phenotypes [7]. Given these complexities, it is critical to investigate whether and to what extent different computable phenotypes of a chronic condition such as T2D affect healthcare utilization prediction.

EHRs are increasingly used for population health management and risk stratification efforts [8]. This is partly due to the increased adoption of EHRs across health providers [9,10], the availability of EHR data on large populations [11], and the existence of unique data types in EHRs such as laboratory results (Lx) which are not commonly available in claims data [12]. Accordingly, computable phenotypes that leverage EHR-specific data types are employed by analysts to identify populations of interest for risk stratification purposes. For example, several computable phenotypes of T2D use medications (Rx) and Lx in addition to diagnostic (Dx) data to identify T2D patients within large population health data repositories [3]. Claims data often lacks Lx data [12], hence assessing the true value of such phenotypes requires the use of EHR data [3]. The impact of variability of computable phenotypes of chronic diseases such as T2D using EHR data, however, is not assessed in the context of population health analytic efforts such as healthcare utilization prediction models.

To overcome the challenges of using EHR data in identifying target T2D patient populations, researchers have used pre-existing phenotyping algorithms to measure and report the sensitivity and specificity of such identification processes [3,13]. However, these approaches alone do not necessarily address the population-level effect of phenotyping on predictive modeling. Currently, little is known about the impact of different computable phenotypes of T2D on healthcare utilization prediction. To address this gap, we assess the impact of T2D phenotypes on identifying different population denominators and their effect on predicting healthcare utilization (i.e., inpatient (IP) and emergency room (ER) admissions) using EHR and claims data. The goal of this study, however, is not to recommend an optimal phenotype for predicting healthcare utilization, as different computable phenotypes of T2D may give rise to different utilization prediction model behaviors. Furthermore, understanding the complexity of each phenotype provides the basis for evaluating outcomes and disease management in different demographics, hence affecting disease surveillance efforts.

## 2. Materials and Methods

### 2.1. Data Source and Study Population

**Figure A1:**
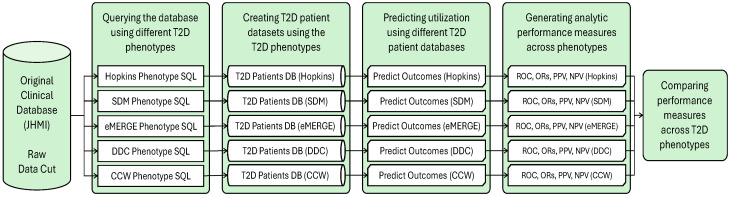
Workflow of the research process: applying the established T2D phenotypes to the original data cut to create different datasets used in the predictive analytic process.

This study used EHR and claims data of approximately 15,338 adult patients with T2D who had at least one outpatient encounter with the Johns Hopkins Medical Institute (JHMI) between 2017 and 2019 and had at least 6 months of continuous insurance enrollment [8]. We required both EHR and claims data for the initial patient population to ensure the availability of all data types for the common T2D phenotypes (i.e., Lx information is collected in EHR data only), and complete measures of IP and ER admissions across all providers (i.e., claims data captured IP/ER admissions outside of the JHMI network) [14]. The initial patient population extracted from JHMI’s data warehouse was identified using any Dx, Rx, or Lx code relevant to T2D, without any conditions required by common computable phenotypes (Figure A1). EHR’s free text was not used to identify additional T2D patients due to limitations of natural language processing techniques (e.g., unrecognized false negatives and potential false positives) and limited practical use to identify large population denominators for routine risk stratification efforts [15].

We used four common T2D computable phenotypes and one locally defined phenotype from Johns Hopkins [16]. The common computable phenotypes of T2D included: (a) Surveillance, Prevention, and Management of Diabetes Mellitus (SUPREME-DM; SDM) [17], (b) eMERGE Northwestern Group (eMERGE) [18], (c) Durham Diabetes Coalition (DDC) [19], and (d) the CMS Chronic Conditions Data Warehouse (CCW) [20]. The SDM, eMERGE, and DDC phenotype definitions include Dx, Rx, and Lx codes. The CCW phenotype only includes Dx codes. Dx codes were converted from ICD-9 to ICD-10 for standardized diagnosis codes [21]. In addition, we converted all Rx codes to RxNorm codes as some phenotypes included criteria using NDC (National Drug Codes) [22].

### 2.2. Variables and Measures

Five T2D computable phenotypes of SDM, CCW, eMERGE, DDC, and Hopkins were used to define the T2D population denominators. Variables needed by these phenotypes were extracted from the collective data represented by the EHR and claims data. Dx (ICD-10) and Rx (RxNorm) codes were extracted from EHR and claims data. Lx (LOINC) codes were extracted from EHR data.

Predictors used for modeling healthcare utilization included demographics (i.e., age, sex, race, ethnicity) and Dx codes. Patients with missing demographic data in both EHR and claims data were excluded from the analysis (less than 1% of the population). Dx codes were summarized using the Charlson comorbidity index [23]. Other predictors included insurance type/status and prior utilization markers, which were extracted from claims data. Model outcomes were IP and ER admissions, which were extracted from claims data to represent healthcare utilization across all providers. Variables selected and models constructed for the analysis followed a validated approach developed previously [8,14].

### 2.3. Statistical Analysis

We constructed concurrent and prospective prediction models using logistic regression for each phenotype using R [24]. Concurrent predictive models apply predictors and outcomes extracted from the same year of the data, while prospective models use predictors occurring a year before the outcomes [8]. We ran 100 model iterations using 70% for training and 30% for testing for each phenotype to measure prediction performance. Our analysis included 10 models per year given the availability of 5 phenotypes and 2 outcomes of interest (i.e., IP and ER). For each phenotype, we calculated odds ratios (ORs), 100-run mean area under the curve (AUC), sensitivity, specificity, positive predictive value (PPV), and negative predictive value (NPV). We also calculated the 95% confidence interval (CI) for each of the measures from the 100 iterations.

This study was reviewed and approved with waivers of informed consent by the IRB committee of the Johns Hopkins School of Public Health (IRB # 00014440).

## 3. Results

**Figure 1:**
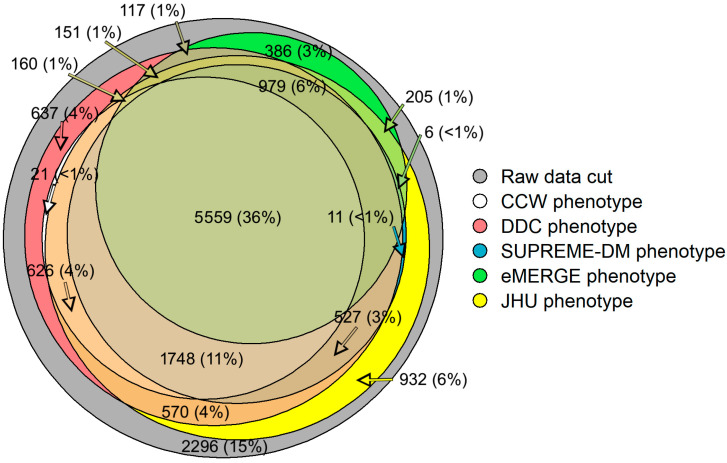
Venn diagram showing the overlap of study populations identified by each computable phenotype using EHR and claims data. Not all counts are shown for visualization purposes.

Our study shows that 36% (n = 5559) of the patient population overlapped across all computable phenotypes of T2D (Figure 1) [16]. Approximately 15% (n = 2296) of the initial population did not fit the characteristics laid out by any of the five phenotypes. Additionally, some of the study populations satisfied the characteristics of only one of the five phenotypes. For example, around 3% (n = 386) of patients with T2D were qualified under the eMERGE phenotype; however, these patients were not identified as having T2D by the other phenotypes.

The average age of the population identified in the raw data cut was approximately 49.5 years of age (Table 1). Age ranged between 51.0 and 52.9 years across the populations identified using different computable phenotypes of T2D. The percentage of females varied between 58.4 and 61.1 across the phenotypes. SDM phenotype identified a T2D population with the highest percentage of Black patients (53.3%), while eMERGE’s population contained the lowest percentage of Black patients (51.7%). The Charlson comorbidity score ranged from 2.48 in the population identified by the Hopkins phenotype to 2.74 for the SDM phenotype. The number of IP admissions was highest in the population identified by the CCW phenotype (average of 3.57 admissions across all years) but lowest for the eMERGE phenotype (2.70 admissions). Population identified by the CCW computable phenotype of T2D also showed the highest ER admissions (average of 3.13 admissions across all years) compared to populations identified by other T2D phenotypes (Table 1).

**Figure A2:**
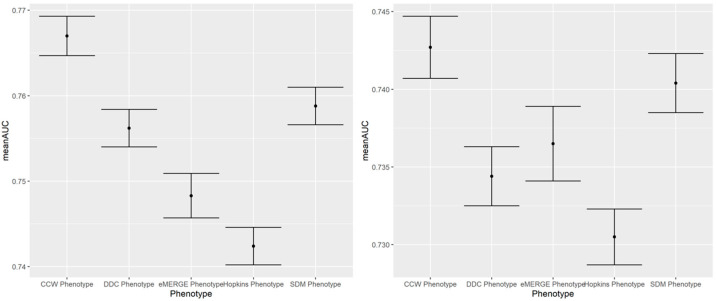
A 100-run mean AUC of 2018 IP (**left**) and ER (**right**) admission prediction using EHR-based predictors and claims-based outcomes of the same year (i.e., concurrent prediction).

**Figure A3:**
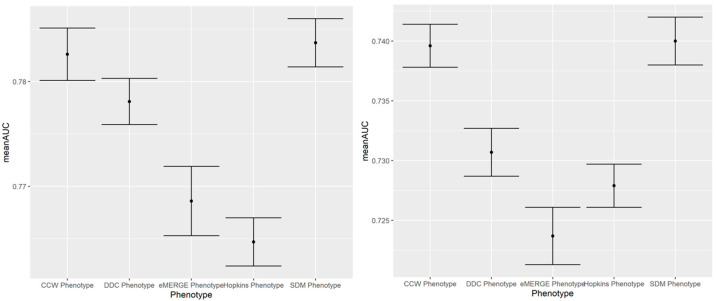
A 100-run mean AUC of 2017 IP (**left**) and ER (**right**) admission prediction using EHR-based predictors and claims-based outcomes of the same year (i.e., concurrent prediction).

Mean AUCs and 95% CIs of the predictive models of IP and ER admissions for each of the phenotypes, along with the PPV and NPV of the models, were measured and compared (Table 2). CCW and SDM had statistically significantly higher mean AUCs (0.7996 and 0.7975) for IP admissions compared to other phenotypes. While CCW and SDM had overlapping CIs, their mean AUCs were statistically significantly higher (0.7371 and 0.7383) for ER admissions compared to other phenotypes. DDC and eMERGE phenotypes showed significant overlaps in their prediction performance for IP admission with AUC CIs of [0.7827, 0.7871] and [0.7831, 0.7885], respectively (Table 2). For ER admissions, the performance of DDC, Hopkins and eMERGE phenotypes had overlapping CIs ([0.7282, 0.7324], [0.7275, 0.7317], and [0.7298, 0.7344]). (Table 2). For both IP and ER admissions, the CCW phenotype had the highest PPVs and lowest NPVs compared to other phenotypes. Results for similar models predicting 2017 and 2018 outcomes were also calculated (Table A1), as well as additional results of 100-run mean AUCs with CIs for 2018 and 2017 by IP and ER across each phenotype (Figure A2 and Figure A3).

**Figure 2:**
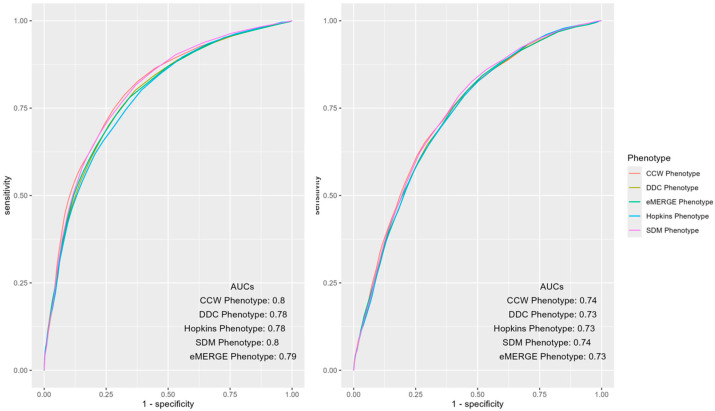
AUC ROC curves of IP (**left**) and ER (**right**) admissions using predictors and outcomes of 2019 across different computable phenotypes of T2D.

ROC curves of the mean AUCs of predictive models of IP and ER admissions in 2019 using various populations identified by the T2D phenotypes were further inspected for notable differences (Figure 2). The overall AUCs across phenotypes were similar for IP and ER admissions; however, differences were observed in certain areas of the curve. For instance, CCW was superior when the false positive rate was less than 0.5, otherwise SDM dominated the other phenotypes.

**Figure 3:**
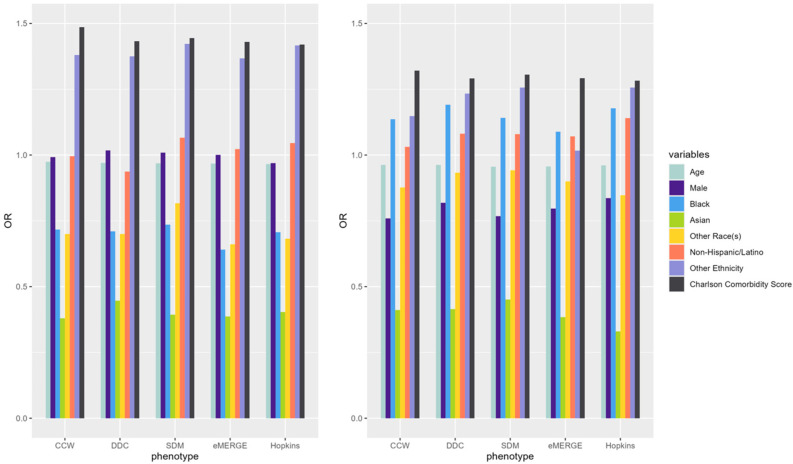
ORs of variables predicting claims-based IP (**left**) and ER (**right**) in 2019 using predictors from the same year (i.e., concurrent prediction) across selected T2D computable phenotypes.

ORs of underlying predictors (e.g., age, male, Black, Asian, Charlson comorbidity score) of IP and ER admissions were similar across the models derived from populations identified by the selected T2D computable phenotypes (Figure 3). Compared to White patients, Black patients had a higher OR than other racial groups across all phenotypes while predicting ER admissions. Males had lower odds of incurring an IP or ER admission. Patients with higher comorbidity scores had higher odds of IP and ER utilization across all phenotypes. Odds ratio CIs for each of the predictors across phenotypes were also calculated (Table A2).

## 4. Discussion

EHRs are increasingly used by healthcare providers for population health analytics and management efforts [8,25,26,27]. Computable phenotypes are often used to identify population denominators that can be used for the development of risk prediction models and prioritization of scarce population-level prevention or intervention resources. However, chronic conditions of interest to population health experts (e.g., T2D) often have multiple computable phenotypes thus affecting the denominator selection, model development, and eventually identification of at-risk populations. Furthermore, uncertainty in selecting proper computable phenotypes can lead to challenges in making appropriate clinical, financial, operational, and strategic decisions. To address these gaps within a population health management context, our analysis attempted to depict the differences in population denominators identified using T2D phenotypes and assess the effect of phenotype selection on developing and evaluating healthcare utilization prediction models using EHR and claims data.

While the goal was not to optimize model performance, our study provides a strong foundation for measuring and comparing the effect of phenotypes on healthcare utilization predictions when using EHR and claims data. As depicted in Figure 1, different phenotype definitions identified different populations (e.g., DDC and Hopkins phenotypes found ~11.5 k T2D patients while CCW and eMERGE phenotypes found ~8 k T2D patients each). The populations identified by each of the T2D phenotypes, as shown in Table 1, showed different compositions (e.g., SDM found a slightly older population with a higher Charlson comorbidity score compared to other phenotypes). These differences could be imperative if such differences affect the predictors or outcomes of interest for clinical research (e.g., enrollment of patients), disease surveillance (e.g., detection of cases), disease management, or prediction of healthcare utilization (e.g., predicting IP and ER admissions). Furthermore, measuring the effect of computable phenotypes in identifying different population denominators may provide opportunities to understand potential disparities in population health outcomes [16].

The study findings showed similarities and differences among mean AUCs (and CIs) of predictive models of IP and ER admissions trained and tested using different population denominators identified by the selected T2D computable phenotypes (Table 2 and Figure 2). Although the AUCs differed modestly across phenotypes, small differences in model performance, when applied across large patient populations, may result in considerable difference in ranking of patients for health utilization outcomes. These changes could impact policy regarding funding allocation or design of preventative strategies for population health management purposes [14]. Additionally, the interpretation of these results could vary depending on the research question and setting. For example, in targeted interventions using an expensive approach for a T2D population [28], resources may be constrained due to the high economic factors associated with it. Although phenotypes had similar performance overall, population health experts, depending on the tradeoffs of false positives and false negatives, may prioritize some phenotypes over others (Figure 2) for IP or ER admissions. CCW may be an appropriate phenotype in this case since it showed the highest mean AUC among all phenotypes. However, it does not leverage Rx and Lx codes to identify a more inclusive denominator of T2D patients using EHR data. Conversely, a surveillance program targeting populations with prediabetes with pamphlets and online resources for education, which has a much lower financial implication and relatively lower possibility of harm, may choose a different set of phenotypes to identify the target population of T2D patients.

IP and ER admission predictors showed similar results across T2D phenotypes with some minor exceptions. As shown in Figure 3, the ORs of age, sex, race, and Charlson comorbidity index were similar across all phenotypes for each of the outcomes. For example, the Black population and the Charlson comorbidity score had ORs well above 1 in predicting ER admissions, while ORs were below 1 for most predictors across all T2D phenotypes when predicting IP admissions. The effect of the Charlson comorbidity score was consistently high regardless of the admission type. These similarities indicate that the overall trends of ORs among underlying predictors of IP and ER admissions did not change across population denominators identified by different T2D computable phenotypes; however, the ORs may slightly change from one phenotype to another. ORs also slightly changed across different years of the data across phenotypes. Further studies are needed to investigate the potential effects of the overall similarity of ORs for all predictors across phenotypes while observing slight differences in the ORs of individual predictors.

We used EHR data as the primary data source of variables used for phenotyping, with Lx only being available in the EHRs, thus making our research particularly practical and applicable to population health efforts undertaken by health systems. We intentionally used claims data as the source of the outcome variables as claims data captures utilization within and outside of a health systems’ network. For example, an admission to a hospital, which is out of a health system’s network, will not be recorded in the health system’s EHR if such information is not being actively exchanged; however, such utilization information will be captured in the patient’s claims data. Despite the advantage of claims data in capturing utilization data, we also conducted a sensitivity analysis using EHR data as the only source of both predictors and outcomes, thus predicting IP or ER admissions to the JHMI health system only. The underlying population used in the sensitivity analysis was much larger (~200 k patients) as availability of claims was no longer a requirement. Nonetheless, the overall results were similar to the results presented in this study. Thus, we found our results to apply to health systems that only have access to EHR data and are mainly interested in predicting healthcare utilization within their network of providers (i.e., IP and ER admissions captured in their EHRs).

**Figure A4:**
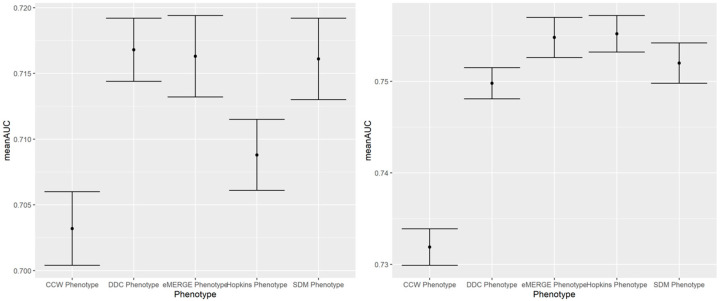
100-run mean AUC of 2019 IP (**left**) and ER (**right**) admission prediction using EHR-based predictors and claims-based outcomes of the same year (i.e., prospective prediction).

**Figure A5:**
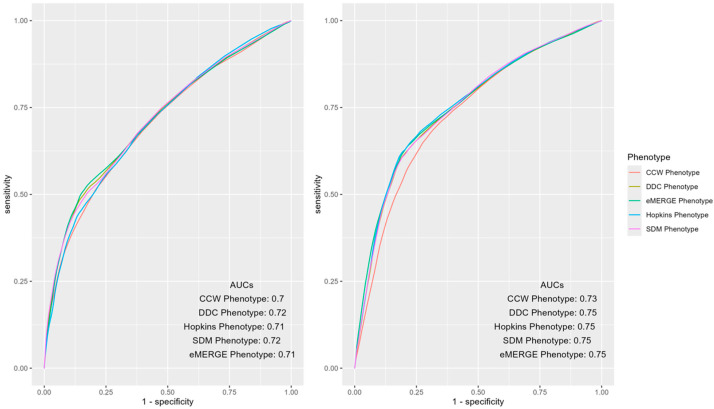
AUC ROC curves of IP (**left**) and ER (**right**) admissions using EHR-based predictors of 2018 and claims-based outcomes of 2019 (i.e., prospective prediction) across different computable phenotypes of T2D.

**Figure A6:**
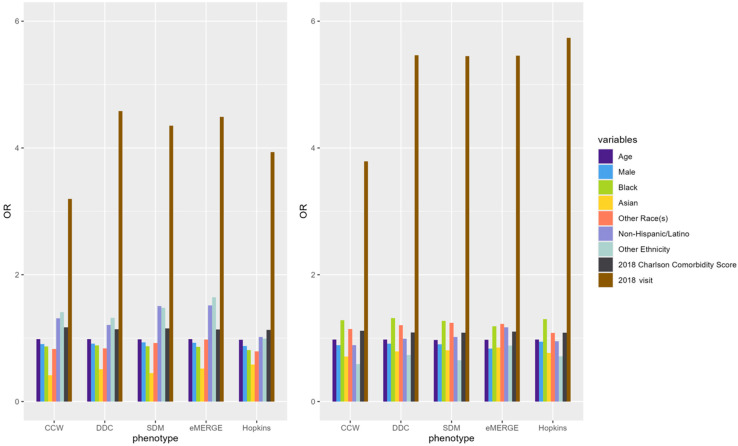
ORs of variables predicting claims-based IP (**left**) and ER (**right**) in 2019 using EHR-based predictors in 2018 (i.e., prospective prediction).

The performance of the prediction models presented in Figure 2 and Figure 3 are based on models trained and tested on the same year of the data (i.e., concurrent predictions). In comparison to prospective models, concurrent models often show higher performance in predicting utilization as predictors are not always temporally arranged before the outcomes of interest [8]. As shown in Table 2, the mean AUCs of the IP and ER prediction models were between 0.73 and 0.80 across all the phenotypes. In contrast to concurrent models, prospective models extract predictors and outcomes from two consecutive years, in which predictors always occur in the year(s) before the outcome. To further examine the generalizability of our findings, we replicated the study to assess the impact of population denominators identified by various T2D computable phenotypes on the performance of prospective models that employ predictors from the previous year to predict IP and ER admissions in the next year (e.g., using 2018 to predict 2019) (Table A3 and Table A4). As shown in Figure A4, Figure A5 and Figure A6, overall performance was lower across prospective models. CCW became the worst-performing phenotype in the prospective prediction task. Deteriorating performance was likely due to the simplicity of the CCW phenotype, missing some patients who should have been included in the denominator for model learning.

Our study had some limitations. First, claims data was available only for a portion of the study population which may lead to potential data biases [16]. Claims data used in this study also lacked Lx data which may limit the comparison of EHR versus claims across phenotypes that use such information in identifying T2D patients. Second, Rx data was not used as a predictor of utilization thus limiting the comorbidity measurement to the Charlson index only. Integrating more sophisticated prediction models that also integrate Rx, such as the Johns Hopkins ACG scores [29], should be investigated in future research. Third, our study may have been impacted when converting Rx codes from NDC to RxNorm; and similarly, when converting Dx codes from ICD-9 to ICD-10. Fourth, the selection of the cut-off value closest to the upper-left corner of the ROC curve to maximize PPV and NPV may not be the best-case scenario for different demographics than our study population [30]. Fifth, our study did not assess outpatient (OP) visits as an outcome because JHMI is a tertiary healthcare center where OP data may be skewed compared to larger health networks that provide primary care. Lastly, although our results indicate that the difference in phenotypes used to identify the population of interest can affect the performance of prediction models in an academic medical center, the size of such an impact should be measured separately for different clinical settings (e.g., community hospitals) or population strata (e.g., across racial groups) [31].

Future research should investigate if certain phenotypes often result in higher predictive performance across different data sources, and if a specific reason is causing such generalizable effects. Future research should also explore the potential racial/ethnic bias of predictive models generated using population denominators identified by different phenotypes [16,31]. This can be achieved upon analysis of racial/ethnic stratification and comparison of populations with T2D across different phenotypes. Another research opportunity could be assessing the trade-offs for the cut-off value of PPV and NPV depending upon various use cases. Lastly, additional studies should analyze the effect of data quality issues (e.g., completeness, accuracy, and timeliness) on identifying population denominators using various T2D computable phenotypes [16].

## 5. Conclusions

Our study utilized EHR and claims data to highlight the assessment of EHR-derived prediction models of IP and ER admissions with claims-based outcomes within each T2D computable phenotype. In summary, our study showed that various computational phenotypes of T2D have a meaningful impact on the identification of T2D patients and the prediction of IP and ED admissions. Findings from our study encourage researchers to understand the variations, robustness, and challenges of each phenotype definition to determine appropriate denominator populations. Additionally, it is critical to comprehend that such variations may impact healthcare utilization predictions to further affect strategic healthcare decisions for populations with T2D.

## Figures and Tables

**Table 1:** Distribution of the study population across each of the computable phenotypes of T2D.

Population Spec	Raw Data	CCW	DDC	SDM	eMERGE	Hopkins	*p*-Value
N	15,338	8410	11,413	9194	7584	11,528	
Age							
Mean (SD)	49.5 (13.2)	51.8 (11.2)	51.4 (12.2)	52.9 (11.1)	52.5 (11.5)	51.0 (12.4)	<0.001
Sex							
Female (%)	9393 (61.2)	5022 (59.7)	6869 (60.2)	5367 (58.4)	4441 (58.6)	7042 (61.1)	<0.001
Male (%)	5943 (38.7)	3387 (40.3)	4542 (39.8)	3826 (41.6)	3143 (41.4)	4484 (38.9)	
Race							
White (%)	5380 (35.1)	2804 (33.3)	3863 (33.8)	3032 (33.0)	2557 (33.7)	3853 (33.4)	0.066
Black (%)	7904 (51.5)	4431 (52.7)	5997 (52.5)	4900 (53.3)	3918 (51.7)	6114 (53.0)	
Asian (%)	826 (5.4)	485 (5.8)	623 (5.5)	508 (5.5)	468 (6.2)	640 (5.6)	
Other (%)	1228 (8.0)	690 (8.2)	930 (8.1)	754 (8.2)	641 (8.5)	921 (8.0)	
Ethnicity							
Hispanic (%)	728 (4.7)	379 (4.5)	532 (4.7)	421 (4.6)	346 (4.6)	532 (4.6)	0.385
Non-Hispanic (%)	13,962 (91.0)	7616 (90.6)	10,350 (90.7)	8322 (90.5)	6881 (90.7)	10,484 (90.9)	
Charlson Comorbidity Score							
Mean (SD)	2.31 (2.47)	2.68 (2.40)	2.53 (2.41)	2.74 (2.40)	2.54 (2.37)	2.48 (2.43)	<0.001
Number of IP Admissions							
Mean (SD)	2.89 (8.74)	3.57 (10.0)	2.88 (8.88)	3.03 (9.28)	2.70 (8.37)	2.81 (8.90)	<0.001
Number of ER Admissions							
Mean (SD)	2.46 (6.86)	3.13 (7.46)	2.45 (6.60)	2.49 (6.42)	2.40 (7.25)	2.54 (7.32)	<0.001

ER: emergency room; IP: inpatient; SD: standard deviation.

**Table 2:** Performance of concurrent prediction models of IP and ER admissions in 2019 across each of the computable phenotypes of T2D.

Outcome Year	Phenotype	Mean AUC [95% CI]	Mean Sensitivity [95% CI]	Mean Specificity [95% CI]	Mean PPV [95% CI]	Mean NPV [95% CI]
IP 2019	CCW	0.7996 [0.7974, 0.8018]	0.7436 [0.7385, 0.7487]	0.7268 [0.7222, 0.7314]	0.3150 [0.3119, 0.3181]	0.9440 [0.9431, 0.9449]
	DDC	0.7849 [0.7827, 0.7871]	0.7242 [0.7188, 0.7296]	0.7209 [0.7161, 0.7257]	0.2629 [0.2603, 0.2655]	0.9503 [0.9495, 0.9511]
	Hopkins	0.7792 [0.7769, 0.7815]	0.7099 [0.7026, 0.7172]	0.7148 [0.7076, 0.7220]	0.2580 [0.2543, 0.2617]	0.9469 [0.9460, 0.9478]
	SDM	0.7975 [0.7950, 0.8000]	0.7279 [0.7222, 0.7336]	0.7343 [0.7285, 0.7401]	0.2793 [0.2758, 0.2828]	0.9506 [0.9498, 0.9514]
	eMERGE	0.7858 [0.7831, 0.7885]	0.7228 [0.7155, 0.7301]	0.7250 [0.7183, 0.7317]	0.2574 [0.2538, 0.2610]	0.9525 [0.9516, 0.9534]
ER 2019	CCW	0.7371 [0.7349, 0.7393]	0.6719 [0.6667, 0.6771]	0.6956 [0.6906, 0.7006]	0.3251 [0.3221, 0.3281]	0.9071 [0.9060, 0.9082]
	DDC	0.7303 [0.7282, 0.7324]	0.6724 [0.6667, 0.6781]	0.6796 [0.6740, 0.6852]	0.2669 [0.2644, 0.2694]	0.9232 [0.9223, 0.9241]
	Hopkins	0.7296 [0.7275, 0.7317]	0.6761 [0.6698, 0.6824]	0.6731 [0.6674, 0.6788]	0.2642 [0.2620, 0.2664]	0.9232 [0.9222, 0.9242]
	SDM	0.7383 [0.7361, 0.7405]	0.6807 [0.6739, 0.6875]	0.6851 [0.6786, 0.6916]	0.2749 [0.2720, 0.2778]	0.9250 [0.9239, 0.9261]
	eMERGE	0.7321 [0.7298, 0.7344]	0.6879 [0.6813, 0.6945]	0.6644 [0.6583, 0.6705]	0.2560 [0.2537, 0.2583]	0.9273 [0.9263, 0.9283]

ER: emergency room; IP: inpatient.

## Data Availability

Restrictions apply to the availability of these data. Data was obtained from the Johns Hopkins Medical Institute (JHMI) and is available from JHMI with permission from the JHMI Data Governance committee.

## Abbreviations

The following abbreviations are used in this manuscript:

AUC	area under the curve
CCW	CMS Chronic Conditions Data Warehouse
CI	confidence interval
DDC	Durham Diabetes Coalition
Dx	diagnosis
EHR	electronic health record
eMERGE	eMERGE Northwestern Group
ER	emergency room
IP	inpatient
JHMI	Johns Hopkins Medical Institute
Lx	laboratory
NDC	National Drug Codes
NPV	negative predictive value
OR	odds ratio
PPV	positive predictive value
Rx	medication
SDM	Surveillance Prevention and Management of Diabetes Mellitus (SUPREME-DM)
T2D	type 2 diabetes

## Appendix A

**Table A1:** Mean AUC, mean PPV and mean NPV with CI for concurrent prediction of IP and ER admission in 2017 and 2018 across phenotypes.

Outcome Year	Phenotype	Mean AUC [95% CI]	Mean Sensitivity [95% CI]	Mean Specificity [95% CI]	Mean PPV [95% CI]	Mean NPV [95% CI]
IP 2018	CCW	0.7670 [0.7647, 0.7693]	0.7013 [0.6961, 0.7065]	0.7142 [0.7092, 0.7192]	0.3417 [0.3384, 0.3450]	0.9191 [0.918, 0.9202]
	DDC	0.7562 [0.7540, 0.7584]	0.6829 [0.6776, 0.6882]	0.7091 [0.7038, 0.7144]	0.2907 [0.2878, 0.2936]	0.9280 [0.9272, 0.9288]
	Hopkins	0.7424 [0.7402, 0.7446]	0.6659 [0.6603, 0.6715]	0.7042 [0.6984, 0.7100]	0.2750 [0.2720, 0.2780]	0.9264 [0.9256, 0.9272]
	SDM	0.7588 [0.7566, 0.7610]	0.6962 [0.6908, 0.7016]	0.7010 [0.6953, 0.7067]	0.2941 [0.2909, 0.2973]	0.9285 [0.9276, 0.9294]
	eMERGE	0.7483 [0.7457, 0.7509]	0.6740 [0.6684, 0.6796]	0.6962 [0.6909, 0.7015]	0.2789 [0.2761, 0.2817]	0.9249 [0.9240, 0.9258]
ER 2018	CCW	0.7427 [0.7407, 0.7447]	0.7034 [0.6988, 0.708]	0.6702 [0.6663, 0.6741]	0.3581 [0.3558, 0.3604]	0.8965 [0.8953, 0.8977]
	DDC	0.7344 [0.7325, 0.7363]	0.6966 [0.6924, 0.7008]	0.6617 [0.6580, 0.6654]	0.2978 [0.2959, 0.2997]	0.9139 [0.9130, 0.9148]
	Hopkins	0.7305 [0.7287, 0.7323]	0.6939 [0.6892, 0.6986]	0.6572 [0.6529, 0.6615]	0.2959 [0.2940, 0.2978]	0.9120 [0.9110, 0.9130]
	SDM	0.7404 [0.7385, 0.7423]	0.7043 [0.6994, 0.7092]	0.6642 [0.6600, 0.6684]	0.3047 [0.3027, 0.3067]	0.9151 [0.9141, 0.9161]
	eMERGE	0.7365 [0.7341, 0.7389]	0.6960 [0.6908, 0.7012]	0.6678 [0.6627, 0.6729]	0.2920 [0.2895, 0.2945]	0.9181 [0.9171, 0.9191]
IP 2017	CCW	0.7826 [0.7801, 0.7851]	0.6860 [0.6813, 0.6907]	0.7628 [0.7578, 0.7678]	0.3433 [0.3392, 0.3474]	0.9311 [0.9302, 0.932]
	DDC	0.7781 [0.7759, 0.7803]	0.6900 [0.6852, 0.6948]	0.7489 [0.7446, 0.7532]	0.2878 [0.2849, 0.2907]	0.9429 [0.9422, 0.9436]
	Hopkins	0.7647 [0.7624, 0.7670]	0.6818 [0.6769, 0.6867]	0.7229 [0.7175, 0.7283]	0.2612 [0.2581, 0.2643]	0.9409 [0.9402, 0.9416]
	SDM	0.7837 [0.7814, 0.7860]	0.7047 [0.6994, 0.7100]	0.7385 [0.7328, 0.7442]	0.2895 [0.2858, 0.2932]	0.9434 [0.9427, 0.9441]
	eMERGE	0.7686 [0.7653, 0.7719]	0.6851 [0.6794, 0.6908]	0.7390 [0.7338, 0.7442]	0.2745 [0.2707, 0.2783]	0.9425 [0.9416, 0.9434]
ER 2017	CCW	0.7396 [0.7378, 0.7414]	0.6770 [0.6733, 0.6807]	0.6774 [0.6742, 0.6806]	0.3996 [0.3974, 0.4018]	0.8689 [0.8678, 0.8700]
	DDC	0.7307 [0.7287, 0.7327]	0.6816 [0.6775, 0.6857]	0.6591 [0.6555, 0.6627]	0.3333 [0.3311, 0.3355]	0.8924 [0.8913, 0.8935]
	Hopkins	0.7279 [0.7261, 0.7297]	0.6757 [0.6712, 0.6802]	0.6585 [0.6544, 0.6626]	0.3313 [0.3292, 0.3334]	0.8904 [0.8893, 0.8915]
	SDM	0.7400 [0.7380, 0.7420]	0.6913 [0.6861, 0.6965]	0.6661 [0.6625, 0.6697]	0.3446 [0.3427, 0.3465]	0.8949 [0.8936, 0.8962]
	eMERGE	0.7237 [0.7213, 0.7261]	0.6711 [0.6651, 0.6771]	0.6632 [0.6580, 0.6684]	0.3190 [0.3167, 0.3213]	0.8960 [0.8947, 0.8973]

**Table A2:** Odds ratios of the model predicting concurrent IP and ER admissions in 2019 across phenotypes.

Phenotype	Variable	IP 2019 Odds Ratio	*p*-Value	ER 2019 Odds Ratio	*p*-Value
CCW	Age	0.975 [0.967, 0.983]	<0.001	0.963 [0.956, 0.969]	<0.001
	Female	Reference Group		Reference Group	
	Male	0.993 [0.831, 1.186]	0.668	0.767 [0.654, 0.901]	0.004
	White	Reference Group		Reference Group	
	Black	0.717 [0.594, 0.865]	0.003	1.140 [0.962, 1.349]	0.183
	Asian	0.382 [0.223, 0.655]	0.001	0.401 [0.242, 0.664]	0.001
	Other Race(s)	0.710 [0.465, 1.084]	0.156	0.888 [0.606, 1.300]	0.557
	Hispanic/Latino	Reference Group		Reference Group	
	Non-Hispanic/Latino	0.971 [0.584, 1.615]	0.701	1.030 [0.652, 1.628]	0.705
	Other Ethnicity	1.341 [0.736, 2.446]	0.386	1.139 [0.656, 1.976]	0.587
	Charlson Comorbidity Score	1.488 [1.440, 1.537]	<0.001	1.322 [1.284, 1.360]	<0.001
DDC	Age	0.970 [0.963, 0.977]	<0.001	0.962 [0.956, 0.968]	<0.001
	Female	Reference Group		Reference Group	
	Male	1.027 [0.871, 1.211]	0.649	0.819 [0.704, 0.952]	0.019
	White	Reference Group		Reference Group	
	Black	0.712 [0.599, 0.846]	0.001	1.184 [1.011, 1.386]	0.070
	Asian	0.445 [0.273, 0.724]	0.003	0.415 [0.255, 0.676]	0.001
	Other Race(s)	0.697 [0.468, 1.039]	0.116	0.935 [0.651, 1.341]	0.661
	Hispanic/Latino	Reference Group		Reference Group	
	Non-Hispanic/Latino	0.933 [0.586, 1.484]	0.670	1.070 [0.697, 1.644]	0.671
	Other Ethnicity	1.346 [0.773, 2.347]	0.347	1.218 [0.723, 2.051]	0.480
	Charlson Comorbidity Score	1.429 [1.389, 1.470]	<0.001	1.294 [1.261, 1.328]	<0.001
eMERGE	Age	0.968 [0.959, 0.976]	<0.001	0.957 [0.950, 0.964]	<0.001
	Female	Reference Group		Reference Group	
	Male	1.007 [0.823, 1.233]	0.652	0.792 [0.660, 0.952]	0.033
	White	Reference Group		Reference Group	
	Black	0.642 [0.518, 0.795]	<0.001	1.101 [0.907, 1.337]	0.393
	Asian	0.393 [0.218, 0.707]	0.003	0.388 [0.220, 0.686]	0.002
	Other Race(s)	0.653 [0.401, 1.061]	0.116	0.893 [0.576, 1.386]	0.595
	Hispanic/Latino	Reference Group		Reference Group	
	Non-Hispanic/Latino	0.991 [0.549, 1.789]	0.731	1.058 [0.619, 1.807]	0.664
	Other Ethnicity	1.312 [0.650, 2.649]	0.465	1.013 [0.525, 1.951]	0.687
	Charlson Comorbidity Score	1.432 [1.383, 1.482]	<0.001	1.294 [1.254, 1.335]	<0.001
Hopkins	Age	0.967 [0.960, 0.973]	<0.001	0.961 [0.955, 0.966]	<0.001
	Female	Reference Group		Reference Group	
	Male	0.977 [0.829, 1.151]	0.646	0.836 [0.719, 0.972]	0.047
	White	Reference Group		Reference Group	
	Black	0.701 [0.592, 0.831]	<0.001	1.165 [0.997, 1.362]	0.089
	Asian	0.390 [0.236, 0.646]	0.001	0.331 [0.197, 0.558]	<0.001
	Other Race(s)	0.684 [0.454, 1.030]	0.103	0.843 [0.578, 1.230]	0.422
	Hispanic/Latino	Reference Group		Reference Group	
	Non-Hispanic/Latino	1.064 [0.655, 1.728]	0.652	1.119 [0.715, 1.751]	0.586
	Other Ethnicity	1.441 [0.809, 2.569]	0.268	1.234 [0.718, 2.123]	0.479
	Charlson Comorbidity Score	1.418 [1.379, 1.458]	<0.001	1.284 [1.252, 1.317]	<0.001
SDM	Age	0.968 [0.960, 0.976]	<0.001	0.956 [0.949, 0.963]	<0.001
	Female	Reference Group		Reference Group	
	Male	1.011 [0.845, 1.209]	0.717	0.767 [0.651, 0.905]	0.005
	White	Reference Group		Reference Group	
	Black	0.726 [0.600, 0.879]	0.004	1.143 [0.958, 1.363]	0.195
	Asian	0.379 [0.213, 0.674]	0.002	0.460 [0.274, 0.774]	0.008
	Other Race(s)	0.819 [0.534, 1.255]	0.403	0.938 [0.629, 1.398]	0.660
	Hispanic/Latino	Reference Group		Reference Group	
	Non-Hispanic/Latino	1.075 [0.635, 1.819]	0.657	1.044 [0.644, 1.694]	0.663
	Other Ethnicity	1.439 [0.772, 2.683]	0.299	1.204 [0.676, 2.145]	0.523
	Charlson Comorbidity Score	1.447 [1.403, 1.493]	<0.001	1.307 [1.270, 1.344]	<0.001

**Table A3:** Odds ratios of the model predicting prospective IP and ER admissions in 2019 across phenotypes.

Phenotype	Variable	IP 2019 Odds Ratio	*p*-Value	ER 2019 Odds Ratio	*p*-Value
CCW	Age	0.986 [0.978, 0.994]	0.002	0.976 [0.970, 0.983]	<0.001
	Female	Reference Group		Reference Group	
	Male	0.907 [0.758, 1.084]	0.339	0.890 [0.765, 1.036]	0.180
	White	Reference Group		Reference Group	
	Black	0.869 [0.720, 1.049]	0.195	1.282 [1.088, 1.511]	0.009
	Asian	0.412 [0.247, 0.689]	0.002	0.707 [0.486, 1.029]	0.102
	Other Race(s)	0.823 [0.527, 1.286]	0.431	1.140 [0.789, 1.646]	0.508
	Hispanic/Latino	Reference Group		Reference Group	
	Non-Hispanic/Latino	1.297 [0.747, 2.255]	0.415	0.885 [0.572, 1.370]	0.569
	Other Ethnicity	1.388 [0.712, 2.709]	0.386	0.587 [0.334, 1.031]	0.094
	Charlson Comorbidity Score	1.172 [1.134, 1.211]	<0.001	1.115 [1.082, 1.149]	<0.001
	Previous Visit in 2018	3.194 [2.662, 3.832]	<0.001	3.786 [3.262, 4.394]	<0.001
DDC	Age	0.983 [0.977, 0.990]	<0.001	0.979 [0.973, 0.985]	<0.001
	Female	Reference Group		Reference Group	
	Male	0.913 [0.770, 1.082]	0.349	0.914 [0.791, 1.055]	0.275
	White	Reference Group		Reference Group	
	Black	0.883 [0.741, 1.053]	0.228	1.315 [1.128, 1.533]	0.002
	Asian	0.506 [0.318, 0.804]	0.009	0.787 [0.550, 1.125]	0.247
	Other Race(s)	0.832 [0.544, 1.271]	0.457	1.197 [0.846, 1.693]	0.368
	Hispanic/Latino	Reference Group		Reference Group	
	Non-Hispanic/Latino	1.197 [0.723, 1.981]	0.518	0.986 [0.657, 1.482]	0.693
	Other Ethnicity	1.293 [0.692, 2.419]	0.469	0.728 [0.428, 1.238]	0.295
	Charlson Comorbidity Score	1.142 [1.109, 1.176]	<0.001	1.088 [1.059, 1.118]	<0.001
	Previous Visit in 2018	4.578 [3.860, 5.430]	<0.001	5.458 [4.750, 6.271]	<0.001
eMERGE	Age	0.983 [0.974, 0.991]	0.001	0.973 [0.966, 0.980]	<0.001
	Female	Reference Group		Reference Group	
	Male	0.927 [0.757, 1.136]	0.507	0.835 [0.702, 0.993]	0.073
	White	Reference Group		Reference Group	
	Black	0.861 [0.695, 1.067]	0.238	1.186 [0.983, 1.431]	0.106
	Asian	0.514 [0.300, 0.880]	0.031	0.848 [0.565, 1.272]	0.454
	Other Race(s)	0.964 [0.585, 1.589]	0.624	1.215 [0.804, 1.837]	0.393
	Hispanic/Latino	Reference Group		Reference Group	
	Non-Hispanic/Latino	1.489 [0.769, 2.886]	0.301	1.159 [0.692, 1.941]	0.555
	Other Ethnicity	1.601 [0.723, 3.545]	0.301	0.865 [0.448, 1.670]	0.596
	Charlson Comorbidity Score	1.136 [1.095, 1.179]	<0.001	1.103 [1.066, 1.141]	<0.001
	Previous Visit in 2018	4.483 [3.641, 5.520]	<0.001	5.450 [4.599, 6.459]	<0.001
Hopkins	Age	0.976 [0.969, 0.982]	<0.001	0.978 [0.972, 0.983]	<0.001
	Female	Reference Group		Reference Group	
	Male	0.874 [0.742, 1.028]	0.148	0.942 [0.817, 1.086]	0.448
	White	Reference Group		Reference Group	
	Black	0.810 [0.686, 0.957]	0.027	1.297 [1.116, 1.507]	0.003
	Asian	0.580 [0.385, 0.874]	0.020	0.766 [0.539, 1.089]	0.178
	Other Race(s)	0.787 [0.523, 1.184]	0.312	1.076 [0.756, 1.533]	0.592
	Hispanic/Latino	Reference Group		Reference Group	
	Non-Hispanic/Latino	1.010 [0.629, 1.622]	0.689	0.942 [0.622, 1.428]	0.646
	Other Ethnicity	0.980 [0.539, 1.784]	0.668	0.704 [0.412, 1.204]	0.263
	Charlson Comorbidity Score	1.131 [1.099, 1.164]	<0.001	1.087 [1.058, 1.117]	<0.001
	Previous Visit in 2018	3.932 [3.342, 4.626]	<0.001	5.733 [5.002, 6.571]	<0.001
SDM	Age	0.982 [0.974, 0.991]	<0.001	0.973 [0.966, 0.979]	<0.001
	Female	Reference Group		Reference Group	
	Male	0.933 [0.775, 1.124]	0.478	0.903 [0.772, 1.057]	0.258
	White	Reference Group		Reference Group	
	Black	0.870 [0.715, 1.059]	0.216	1.271 [1.070, 1.510]	0.016
	Asian	0.444 [0.257, 0.766]	0.006	0.802 [0.538, 1.195]	0.334
	Other Race(s)	0.917 [0.575, 1.463]	0.650	1.233 [0.839, 1.812]	0.348
	Hispanic/Latino	Reference Group		Reference Group	
	Non-Hispanic/Latino	1.485 [0.814, 2.709]	0.249	1.011 [0.636, 1.607]	0.681
	Other Ethnicity	1.445 [0.699, 2.986]	0.385	0.643 [0.354, 1.168]	0.205
	Charlson Comorbidity Score	1.153 [1.116, 1.192]	<0.001	1.084 [1.052, 1.118]	<0.001
	Previous Visit in 2018	4.346 [3.594, 5.255]	<0.001	5.443 [4.664, 6.352]	<0.001

**Table A4:** Mean AUC, mean PPV and mean NPV with CI for prospective prediction of IP and ER admission in 2019 across phenotypes.

Outcome Year	Phenotype	Mean AUC [95% CI]	Mean Sensitivity [95% CI]	Mean Specificity [95% CI]	Mean PPV [95% CI]	Mean NPV [95% CI]
IP 2019	CCW	0.7032 [0.7004, 0.7060]	0.6254 [0.6186, 0.6322]	0.6775 [0.6692, 0.6858]	0.3105 [0.3066, 0.3144]	0.8871 [0.8860, 0.8882]
	DDC	0.7168 [0.7144, 0.7192]	0.6244 [0.6167, 0.6321]	0.6914 [0.6818, 0.7010]	0.2738 [0.2692, 0.2784]	0.9093 [0.9083, 0.9103]
	Hopkins	0.7088 [0.7061, 0.7115]	0.6368 [0.6284, 0.6452]	0.6673 [0.6583, 0.6763]	0.2825 [0.2787, 0.2863]	0.9004 [0.8991, 0.9017]
	SDM	0.7161 [0.713, 0.7192]	0.6342 [0.6253, 0.6431]	0.6790 [0.6691, 0.6889]	0.2658 [0.2614, 0.2702]	0.9114 [0.9102, 0.9126]
	eMERGE	0.7163 [0.7132, 0.7194]	0.6051 [0.5960, 0.6142]	0.7280 [0.7159, 0.7401]	0.2931 [0.2860, 0.3002]	0.9107 [0.9096, 0.9118]
ER 2019	CCW	0.7319 [0.7299, 0.7339]	0.6707 [0.6667, 0.6747]	0.7068 [0.7026, 0.7110]	0.5371 [0.5343, 0.5399]	0.8093 [0.8078, 0.8108]
	DDC	0.7498 [0.7481, 0.7515]	0.6583 [0.654, 0.6626]	0.7533 [0.7484, 0.7582]	0.5034 [0.4993, 0.5075]	0.8537 [0.8525, 0.8549]
	Hopkins	0.7552 [0.7532, 0.7572]	0.6714 [0.6669, 0.6759]	0.7532 [0.7484, 0.7580]	0.5161 [0.5122, 0.5200]	0.8546 [0.8534, 0.8558]
	SDM	0.7520 [0.7498, 0.7542]	0.6527 [0.6488, 0.6566]	0.7595 [0.7554, 0.7636]	0.5047 [0.5009, 0.5085]	0.8539 [0.8526, 0.8552]
	eMERGE	0.7548 [0.7526, 0.7570]	0.6621 [0.6575, 0.6667]	0.7624 [0.7574, 0.7674]	0.5057 [0.5014, 0.5100]	0.8607 [0.8594, 0.8620]
